# The effectiveness of integrating clinical pharmacists within general practice to optimise prescribing and health outcomes in primary care patients with polypharmacy: A protocol for a systematic review

**DOI:** 10.12688/hrbopenres.12966.2

**Published:** 2020-03-12

**Authors:** Aisling Croke, Oscar James, Barbara Clyne, Frank Moriarty, Karen Cardwell, Susan M. Smith

**Affiliations:** 1 Department of General Practice, Royal College of Surgeons in Ireland, Dublin, Ireland; 2 NI Centre for Pharmacy Learning & Development, Queens University, Belfast, UK

**Keywords:** Systematic review, Polypharmacy, Multimorbidity, Pharmaceutical Services, Pharmacist, Primary Care

## Abstract

**Introduction: **Coordinating prescribing for patients with polypharmacy is a challenge for general practitioners. Pharmacists may improve management and outcomes for patients with polypharmacy. This systematic review aims to examine the clinical and cost-effectiveness of pharmacist interventions to optimise prescribing and improve health outcomes in patients with polypharmacy in primary care settings.

**Methods: **The review will be reported using the PRISMA guidelines. A comprehensive search of 10 databases from inception to present, with no language restrictions will be conducted. Studies will be included where they evaluate the clinical or cost-effectiveness of a clinical pharmacist in primary care on potentially inappropriate prescriptions using validated indicators and number of medicines. Secondary outcomes will include health related quality of life measures, health service utilisation, clinical outcomes and data relating to cost effectiveness. Randomised controlled trials, non-randomised controlled trials, controlled before-after, interrupted-time-series and health economic studies will be eligible for inclusion.

Titles, abstracts and full texts will be screened for inclusion by two reviewers. Data will be extracted using a standard form. Risk of bias in all included studies will be assessed using the Effective Practice and Organisation of Care (EPOC) criteria. Economic studies will be assessed using the Consensus Health Economic Criteria (CHEC) list as per the Cochrane Handbook for critical appraisal of methodological quality. A narrative synthesis will be performed, and the certainty of evidence will be assessed using the Grading of Recommendations Assessment, Development and Evaluation (GRADE) criteria. Where data support quantitative synthesis, a meta-analysis will be performed.

**Discussion: **This systematic review will give an overview of the effectiveness of pharmacist interventions to improve prescribing and health outcomes in a vulnerable patient group. This will provide evidence to policy makers on strategies involving clinical pharmacists integrated within general practice, to address issues which arise in polypharmacy and multimorbidity.

**PROSPERO Registration:** CRD42019139679 (28/08/19)

## Introduction

Patients with multimorbidity and polypharmacy have complex health needs. Polypharmacy has commonly been defined as being on five or more medications, however recent studies have highlighted the rising prevalence of major polypharmacy defined as being on 10 or more medications ^2^. Polypharmacy increases the risk of potentially inappropriate prescribing (PIP) and treatment burden in this patient cohort ^3^. PIP is described as potential sub-optimal prescribing and is typically identified using screening tools such as the Screening Tool of Older People’s Prescriptions (STOPP)-Screening Tool to Alert to Right Treatment (START) criteria ^4^, Beers criteria ^5^ or prescribing appropriateness indices ^6^. Medicines optimisation is a broad concept adopted for this systematic review which includes the identification and rectification of potentially inappropriate prescribing as defined by validated screening tools. This is in line with the definition adopted by the NICE guidelines ^7^ on medicines optimisation. “A person-centred approach to safe and effective medicines use, to ensure people obtain the best possible outcomes from their medicines”. Medication optimisation can also encompass deprescribing, which is defined as the process of withdrawal of an inappropriate medication, supervised by a health care professional ^8^. Polypharmacy is also associated with adverse drug events, which may create significant cost to both the healthcare system/health service and patients ^9^. An Irish study conducted in 2010 found that an estimated 36% of adult patients over the age of 70 had at least one PIP event. This resulted in an additional €45 million in healthcare expenditures ^10^. “Irrespective of definition or population age, polypharmacy is a major health issue with increasing worldwide prevalence ^11^”.

Integration of clinical pharmacists within general practices may be an effective way to address PIP, and implement deprescribing through medication reviews and address challenges with general practice workload ^12^. Evidence suggests that clinical pharmacists improve the quality and safety of prescribing ^12^, and that non-dispensing pharmacists integrated into the primary care setting add value to patient-centred clinical pharmacy services ^13^. Heterogeneity of outcomes reported in systematic reviews tend to make meta-analysis challenging and thus it is unclear whether such interventions can result in clinically significant improvements in patient outcomes. There is evidence to suggest that clinical pharmacist review of medications and pharmacist-physician collaboration results in cost avoidance in the hospital setting, and that such interventions are cost-effective ^14^. However, there is limited evidence to date surrounding the cost-effectiveness of clinical pharmacists integrated within general practice to optimise medications and health outcomes in primary care ^12, 14^. Previous systematic reviews cite heterogeneity in terms of role, and duration and frequency of intervention as a limitation. They also focussed on interventions where the clinical pharmacist and general practitioner (GP) were geographically co-located. This systematic review will differ in that it focuses on patients with multimorbidity and polypharmacy, without a focus on specific conditions. Previous reviews included studies which were single-condition focused, for example diabetes, hypertension, dyslipidaemias, heart failure or depression ^12, 13^. As this review intends to analyse studies relevant to polypharmacy and multimorbidity these studies will not be reported unless they include information on degree of polypharmacy or other morbidities. It will include studies that involve remote clinical pharmacist and GP integration, provided there is on-going collaboration and clear evidence of working together to improve patient outcomes.

The overall aim of this systematic review is to examine the effectiveness of interventions involving the integration of clinical pharmacists within general practice, to improve prescribing practices and health outcomes in primary care settings. We will also examine the cost-effectiveness of such interventions.

### Review questions

What is the effectiveness of interventions integrating clinical pharmacists within general practice on medicines optimisation and health outcomes in adult patients with polypharmacy in comparison to usual care?

Is integrating clinical pharmacists within general practice, to improve medicines optimisation and health outcomes in adult patients with polypharmacy, cost-effective?

## Methods

The systematic review will be conducted in line with the PRISMA guidelines ^15^. The protocol will be reported in line with the PRISMA-P guidelines ^16^.

### Eligibility criteria

***Participants/population.*** Patient participants must be adult patients aged 18 years and over in the primary care setting with polypharmacy as defined by study author. Patients will be excluded if they are younger than 18 years old. Studies must have a majority of patients (≥80%) identified as having polypharmacy (using any definition). The generally accepted definition is five or more routine medicines ^2^.

Clinical pharmacists participating in the intervention must be involved in medicines optimisation roles and integrated physically or remotely as per the definition below, within the primary care setting. The definition of primary care for this review will be; a system which is “integrated, easy to access, health care services by clinicians who are accountable for addressing a large majority of personal health care needs, developing a sustained and continuous relationship with patients, and practicing in the context of family and community” ^17^. Pharmacist interventions in a nursing home, secondary or tertiary care setting will be excluded.

Nursing homes are defined as;

- “a facility that provides 24-hour functional support for people who require assistance with activities of daily living (ADLs) or instrumental activities of daily living (IADLs) and have identified health needs
- may or may not be staffed with health care professionals
- provides long-term care and/or rehabilitation as part of hospital avoidance or to facilitate early hospital discharges
- does not function as a hospital ward and is not hospital-based
- may play a role in providing palliative and/or hospice care at end of life” ^18^.

Nursing homes are also known as care homes, long term care facilities and aged-care facilities. Nursing care can be provided on a 24-hour basis in these types of homes. Residential homes, assisted-living, and supportive care facilities provide personal care to residents ^19^. Residents in nursing homes typically require assistance with daily living due to identified health needs but do not require hospital care ^18^.

If the setting is unclear, study authors will be contacted to determine the level of access to care the patients had. If confusion persists after contacting study authors the study will be discussed with the systematic review team and a consensus will be reached.

Integration will be defined as per the framework adapted from Walshe and Smith ^20^. The framework consists of six dimensions; organisational, informational, clinical, financial, functional and normative. Four dimensions of the framework map to the inclusion and exclusion criteria of the review;

1. The organisational dimension will involve looking at whether the pharmacist is physically co-located with the GP or if the intervention is remote but encompassed within the same network.
2. The informational dimension refers to integration and access of clinical patient systems.
3. The clinical dimension will encompass care delivery to patients and communication with GPs.
4. The functional dimension allows for the capture of other actions taken by pharmacists integrated within GP settings such as medicines education or administrative support.

Data will be extracted where available on the other dimensions;

5. It is anticipated that the financial dimension will not be measureable as most interventions are externally funded.
6. Normative dimensions will look at the design of interventions in terms of shared goals and visions of activities involved and desired outcomes.

***Intervention/exposure.*** To be eligible for inclusion, studies must involve a clinical pharmacist optimising medication for patients in a primary care setting through a variety of services. Interventions can be targeted at patient or prescriber behaviours. The relationship between pharmacist and GP can be conducted in a co-located setting or remotely where the pharmacist is not in the same geographical location as the GP. The relationship must be continued for the duration of the intervention. This relationship will be defined as a collaborative relationship where “health care professionals assume complementary roles and cooperatively working together, sharing responsibility for problem-solving and making decisions to formulate and carry out plans for patient care” ^21^. Contact can be face to face, virtual, by telephone or via an online forum once the contact is in real-time.

‘Once-off’ interventions where the clinical pharmacist does not maintain a relationship with the GP will be excluded. Such interventions will demonstrate a single instance of unidirectional communication of a medication review issue to the GP (e.g. sending a fax or email) with no collaborative follow-up. We will exclude studies with interventions only targeting a single condition unless the intervention addresses all medicines for the patients, not only those medications which are condition specific.

***Comparator/control.*** Usual care in primary care setting.

***Types of studies.*** We will include randomised controlled trials (RCTs), non-randomised controlled trials (nRCTs), controlled before-after (CBA), and interrupted-time-series (ITS) studies using Cochrane Effective Practice and Organisation of Care (EPOC) study design criteria ^22^. Health economic studies including comparative resource use studies and economic evaluations (cost-effectiveness analysis, cost-utility analysis, cost-minimisation analysis and cost-benefit analysis) will also be included.

***Setting.*** Only studies in a primary care setting will be included.

### Outcomes

***Main outcomes.*** Potentially inappropriate or high risk prescriptions as reported by included studies. Studies may report potentially inappropriate or high risk prescriptions using screening tools such as; STOPP/START ^4^ and Beers criteria ^5^ (explicit criteria), or the Medicines Appropriateness Index ^23^ and Prescribing Appropriateness Index ^24^ (implicit criteria).

Number of medicines. The number of medicines as reported by included studies. The definition of this may vary across studies (e.g. some may use the number of repeat medicines), however where possible we will use the number of medicines including acute and repeat prescription medicines.

***Additional outcomes.***

- Patient reported outcomes measures (PROMs): For example, Health Related Quality of Life measured using standardised questionnaires such as EQ-5D, SF-12, SF-36.
- Adverse events or harms, for example measured using the adverse drug withdrawal reaction scale ^25^.
- Health service utilisation (including GP visits, emergency department (ED) visits, outpatient clinic attendances, inpatient admissions, other healthcare professional appointments).
- Relevant clinical physical outcomes would include measures such as blood pressure, glycosylated haemoglobin as a measure of blood glucose control, serum/blood cholesterol measurement and mortality.
- Mental health outcomes such as depression can be captured using screening tools such as the Warwick Edinburgh Mental wellbeing scale, the Hospital Anxiety and Depression Scale or the Beck Depression Inventory.

These lists are not exhaustive and heterogeneity between studies in terms of outcomes reported is anticipated.

- Economic evaluations;
  ○ Direct costs
  ○ Incremental cost-effectiveness ratio (ICER)
  ○ Cost per unit of effect
  ○ Quality-adjusted life years (QALYs) and disability-adjusted life years (DALYs)
  ○ Cost differences (in measured in cost-benefit / cost-minimisation analysis)
  ○ Other economic measures where data available.

### Search strategy

The following databases will be searched from inception to October 2019; PubMed, Cochrane Library, Cochrane Central Register of Controlled Trials, EMBASE, Web of Science, SCOPUS, Lilacs and CINAHL.

For the cost-effectiveness evaluation the following databases will be searched: NHS Economic Evaluations Database (NHS EED) and the Health Technology Assessment (HTA) database (searchable from the University of York Centre for Reviews and Dissemination site). For both PubMed and EMBASE an economic filter will be applied.

The search strategy for PubMed will include a combination of keywords and MeSH terms for broad concepts of pharmacy and primary health care. This strategy will be adapted for different databases and terms for cost-effectiveness will be included for the economic evaluation component. A review of reference lists of included studies will also be performed. No language limits will be applied. Search strategy is available as extended data ^26^.

***Data management.*** All citations will be downloaded and stored in EndNote reference manager, Version 8. Rayyan software will be used for the title and abstract screening step ^27^.

***Study selection.*** Titles will be screened for clearly ineligible studies by one researcher (AC). Titles and abstracts will be independently screened by two members of the review panel (AC and OJ) to identify studies that potentially meet inclusion criteria outlined above, following duplication removal. Studies which do not meet the inclusion criteria will be excluded at this stage. If studies are unclear or if they meet inclusion criteria, they will be selected for full text review. Where disagreement arises between reviewers, a third reviewer will be consulted (FM). A PRISMA flow chart will be used to display the flow of identified studies through the review.

## Data extraction

Two reviewers (AC and OJ) will use a standardised, pre-piloted form (see extended data ^26^) to perform data extraction of the following information: name of first author, year of publication, country of publication, study setting; study population and participant demographics, intervention details and design, control setting details, recruitment and study completion rates, outcomes and times of measurement.

Economic evaluation data will be extracted as per the Health Information and Quality Authority (HIQA) guidelines for interpretation of economic evaluations in Ireland;

- Study question, population, intervention, comparator and setting
- Modelling methods
- Sources and quality of clinical data
- Cost data
- Resource usage
- Study outcomes
- Methods for dealing with uncertainty.

Where disagreement arises between reviewers, a third reviewer will be consulted (SS or FM).

### Quality assessment

Studies will be included if they meet all inclusion criteria irrespective of quality. The risk of bias in all included effectiveness studies will be assessed using standard EPOC criteria ^22^ (EPOC 2015) including the following domains: allocation (sequence generation and concealment); baseline characteristics; incomplete outcome data; contamination; blinding; selective outcome reporting; and other potential sources of bias. Publication bias will be assessed using a funnel plot if ten or more studies are identified.

The economic evaluation studies will be assessed for methodological quality using the CHEC list as per the Cochrane Handbook for critical appraisal of methodological quality ^28^.

***Assessing the quality of the body of evidence.*** The certainty of evidence for each outcome will be assessed, where appropriate, using the Grading of Recommendations Assessment, Development and Evaluation (GRADE) criteria and GRADEPro software ^29^.

### Strategy for data synthesis

A narrative synthesis is anticipated for effectiveness studies. Similarly, for the economic evaluations a narrative synthesis is anticipated and results displayed in tables.

A statistician will advise if there are sufficient data to support meta-analysis. RevMan 5.3 ^30^ or Stata version 15 ^31^ software will be used. A random effects model will be employed to account for between-study heterogeneity. As per the Cochrane Handbook ^32^ the chi-square test will assess heterogeneity between individual studies. An I ^2^ statistic will assess the impact of heterogeneity on meta-analysis where I ^2^ value greater than 75% indicates significant heterogeneity.

### Analysis of subgroups

Subgroup analysis will be performed if adequate studies exist for interventions and outcomes.

Subgroup analysis will be based on age of patients (18–65 years vs 65 or older), degree of polypharmacy (<10 vs 10 or more) and location of intervention (co-located vs remote location), where data allow. The effect of each individual study on the overall estimates of effect size and determinants will be assessed using sensitivity analyses.

### Dissemination of information

The review will be published in a relevant peer reviewed journal, reported using the PRISMA guidelines. The review will also be presented at a relevant conference and disseminated to policy-makers, patients, and the public.

### Study status

Database searches have been completed and title and abstract screening is currently underway.

## Discussion

This systematic review will give an overview of the effectiveness of interventions involving clinical pharmacist integration within general practice, to improve prescribing and health outcomes in patients with polypharmacy and multimorbidity. We will focus on interventions where the clinical pharmacist and GP work collaboratively to improve patient outcomes, whether co-located or remotely. It is intended that this will address patient populations that live in geographically isolated regions in tandem with patients who live in more geographically connected settings.

This systematic review can contribute to the evidence base for managing multimorbidity. Good quality evidence is required to develop guidelines directed at such complex patients and interventions have been focused on the patients who use clinical services most in recent years ^17^. This review focuses on patients with polypharmacy, a factor in treatment burden for patients with multimorbidity. By addressing this issue in the primary care setting this may improve patient outcomes.

This will provide evidence to policy makers on strategies involving pharmacists integrating within general practice settings to address issues which arise in patients with polypharmacy in a primary care setting.

## Data availability

### Underlying data

No data are associated with this article

### Extended data

Open Science Framework: The effectiveness of integrating clinical pharmacists within general practice to optimise prescribing and health outcomes in primary care patients with polypharmacy: A protocol for a systematic review. https://doi.org/10.17605/OSF.IO/38CU5 ^26^

This project contains the following extended data:

- Data extraction template.xlsx (Excel file containing the data extraction sheet for the study)
- PubMed Search Strategy.docx (Word document containing the PubMed search strategy)

### Reporting guidelines

PRISMA-P checklist for “The effectiveness of integrating clinical pharmacists within general practice to optimise prescribing and health outcomes in primary care patients with polypharmacy: A protocol for a systematic review” https://doi.org/10.17605/OSF.IO/38CU5 ^26^

Data are available under the terms of the Creative Commons Zero “No rights reserved” data waiver (CC0 1.0 Public domain dedication).
