# Supplementary material for: The effectiveness of integrating clinical pharmacists within general practice to optimise prescribing and health outcomes in primary care patients with polypharmacy: A protocol for a systematic review
Source: HRB Open Res. 2020 Mar 12;2:32. Originally published 2019 Nov 19. [Version 2] doi: 10.12688/hrbopenres.12966.2 (PMC7140766; doi:10.12688/hrbopenres.12966.2)
Supplement: Peer review history

# Peer review: Reviewer response for version 2

**DOI:** 10.21956/hrbopenres.14102.r27235

**Published:** 2020-03-19

**Authors:** Nagham J. Ailabouni

**Affiliations:** 1 School of Pharmacy, Department of Pharmacy, University of Washington, Seattle, WA, USA; 2 School of Pharmacy & Medical Sciences, University of South Australia, Adelaide, Australia

Thank you for addressing my comments and revising your manuscript. I am satisfied with the changes made and believe that your response provided added clarity. I have no further comments and I look forward to reading the results of this systematic review.

I confirm that I have read this submission and believe that I have an appropriate level of expertise to confirm that it is of an acceptable scientific standard.

# Peer review: Reviewer response for version 1

**DOI:** 10.21956/hrbopenres.14050.r27008

**Published:** 2020-02-03

**Authors:** Rupert A. Payne

**Affiliations:** 1 Bristol Medical School, Population Health Sciences, Centre for Academic Primary Care, University of Bristol, Bristol, UK

This is a generally clear, well-written protocol for a systematic review looking at the effectiveness of clinical pharmacists integrated within general practice for polypharmacy management. Overall, the methodology is sound, and the proposed review will hopefully provide a useful appraisal of the evidence.

 Additional clarity could be provided around the type of clinical pharmacist in the abstract methods. The title specifically mentions integration of the pharmacist, but I think this should also be explicit in the abstract methods.

 Similarly, it may be useful to expand on the definition used for integration, and to perhaps give more details regarding how the Walshe and Smith framework was operationalised, and perhaps clarifying for the reader whether all six dimensions are required without the reader having to refer to the source reference.

 Further minor points:

- Clarify whether "nursing home" exclusion includes residential or other supportive care environments that don't specifically include nursing, and how situations where the setting is unclear will be dealt with.
- Outcomes - "number of medicines" should ideally be defined (although likely to vary across studies) - will it include both acute and repeat prescriptions, or account for length of treatment (e.g. chronic vs. one-off drugs)?
- The additional outcome "clinical outcomes [physical and mental health]" is very vague - some examples might help clarify what you mean by these. In particular, will they all be some sort of quantitative scale, or will be include discrete events of some description?

I confirm that I have read this submission and believe that I have an appropriate level of expertise to confirm that it is of an acceptable scientific standard.

# Response

**Published:** 2020-03-02

**Authors:** Aisling Croke

**Affiliations:** Royal College of Surgeons in Ireland, Ireland

**Question 1**

**Additional clarity could be provided around the type of clinical pharmacist in the abstract methods. The title specifically mentions integration of the pharmacist, but I think this should also be explicit in the abstract methods.**

Author response:

The American College of Clinical Pharmacy (ACCP) defines clinical pharmacy as a ‘health science discipline in which pharmacists provide patient care that optimises medication therapy, and promotes health, and disease prevention to ensure optimal patient outcomes’ (1). Other studies have cited ‘non-dispensing pharmacists’ (2, 3) but this review cites ‘clinical pharmacist’ to capture remote interventions which may involve ‘dispensing pharmacists’. We have added text to the abstract methods on page 2 to address this comment. 

**Question 2**

**Similarly, it may be useful to expand on the definition used for integration, and to perhaps give more details regarding how the Walshe and Smith framework was operationalised, and perhaps clarifying for the reader whether all six dimensions are required without the reader having to refer to the source reference.**

Author response:

Thank you for highlighting this point. We have added text to the eligibility criteria on page 5 to clarify. 

**Further minor points:**

**Question 3**

**Clarify whether "nursing home" exclusion includes residential or other supportive care environments that don't specifically include nursing, and how situations where the setting is unclear will be dealt with.**

Author response:

A definition of nursing homes has been added to the eligibility criteria on page 4, we also outline how situations where the setting is unclear will be dealt with. 

**Question 4**

**Outcomes - "number of medicines" should ideally be defined (although likely to vary across studies) - will it include both acute and repeat prescriptions, or account for length of treatment (e.g. chronic vs. one-off drugs)?**

Author response:

A definition has been added to the main outcomes section on page 6. 

**Question 5**

**The additional outcome "clinical outcomes [physical and mental health]" is very vague - some examples might help clarify what you mean by these. In particular, will they all be some sort of quantitative scale, or will be include discrete events of some description?**

Author response:

We have addressed this as outlined in our response to the previous reviewer’s comments and have added clarifications around these additional outcomes on page 6.

***References:***

*​​​​​​1. ACCP. DEFINITION OF CLINICAL PHARMACY accp.com [Available from: https://www.accp.com/stunet/compass/definition.aspx.*

*2. Tan EC, Stewart K, Elliott RA, George J. Pharmacist services provided in general practice clinics: a systematic review and meta-analysis. Res Social Adm Pharm. 2014;10(4):608-22.*

*3. Hazen ACM, de Bont AA, Boelman L, Zwart DLM, de Gier JJ, de Wit NJ, et al. The degree of integration of non-dispensing pharmacists in primary care practice and the impact on health outcomes: A systematic review. Res Social Adm Pharm. 2018;14(3):228-40.*

# Peer review: Reviewer response for version 1

**DOI:** 10.21956/hrbopenres.14050.r27007

**Published:** 2020-01-03

**Authors:** Nagham J. Ailabouni

**Affiliations:** 1 School of Pharmacy, Department of Pharmacy, University of Washington, Seattle, WA, USA; 2 School of Pharmacy & Medical Sciences, University of South Australia, Adelaide, Australia

Thank you for the opportunity to review this work. This is an area that requires more attention and the results of this systematic review may have positive consequences that promote increased utilisation of pharmacists in primary care settings to review and optimise medication regimens whilst reducing inappropriate medication use. I look forward to reading the findings of this systematic review. 

**Major Comments**

 Methods:

- Main outcomes: Will all of the listed different medication appropriateness criteria (explicit: STOPP/START, Beer’s criteria and implicit: MAI etc.) be used to assess the medication changes of each pharmacist intervention study included in this systematic review? Can the authors please comment on what will determine the use of explicit vs implicit or the use of both explicit and implicit criteria to assess the outcomes of the included studies?
- Additional Outcomes: More clarity is needed regarding physical and mental clinical outcomes that will be used as secondary outcome measures. Please list a few specific measures as examples. What are some key clinical outcomes that would be important to consider? (e.g. Falls? Mortality? Blood Pressure? etc.)
- Since the “number of medicines” is mentioned as a major outcome of this systematic review and the introduction discusses deprescribing, is the intention to focus on the reduction of the number of medicines through pharmacist medication/deprescribing medication reviews and reduction of prescribing of high-risk medications that are inappropriately prescribed, or will potential prescription omissions as defined by the START criteria also be applied? It seems the authors intend on the later since medicines optimisation is mentioned under the heading of ‘Review questions’ and in the title of the study. Perhaps defining the concept of optimising medicines as reducing potentially inappropriate prescribing/sub-optimal prescribing via reducing potentially inappropriate medications (PIMs) as defined by Beer’s and STOPP and potential prescription omissions (PPOs) defined by START in the introduction might provide a clearer preface for the main goal of the systematic review.
- Analysis of subgroups: Inappropriate polypharmacy and inappropriate prescribing is a common problem in older adults. Can the authors please comment on why the cut off chosen here is patients aged 65 years old? For example, is there enough evidence for cost-effectiveness of pharmacist interventions for this age population? Also, if studies that are purely older adult focused (i.e. 65 years and older) are meant to be excluded and are outside the scope of this intended systematic review, please list this explicitly under eligibility/exclusion criteria.

**Minor Comments:**

 Abstract:  

- Methods: Can the authors please clarify what is meant by ‘validated indicators’? Is STOPP/START, Beer’s criteria etc. what is meant? If so, please use screening tools or criteria to assess medication appropriateness instead. Please change in the remainder of the protocol for consistency.

Introduction:

- Lines 2-3: “Polypharmacy has commonly been defined as being on five or more medications, however recent studies have highlighted the rising prevalence of more significant polypharmacy defined as being on 10 or more medications”. Whilst I agree with the content of this statement, I would recommend to:

1. Refer to more significant polypharmacy (i.e. being prescribed or taking 10 or more medications) as hyperpolypharmacy or excessive polypharmacy as described in the literature.
2. Please clarify that polypharmacy overall regardless of which type is on the rise in different age populations.

- Line 3: The referenced article does not mention the term ‘complex polypharmacy’, but rather the complex treatment burden associated with treating comorbidities. Please rephrase to ‘polypharmacy’ to remain consistent with the rest of the introduction.
- I would suggest combining the second paragraph beginning with “Polypharmacy is also associated with adverse drug events…” with the earlier paragraph and consolidate main concepts related to the negative consequences of polypharmacy and how polypharmacy is associated with an increased likelihood of experiencing inappropriate polypharmacy.

- Line 11: Please define deprescribing.

- Line 21: Could the authors please clarify what is meant by this systematic review will not focus on specific conditions? If previous systematic reviews focused on specific conditions (e.g. Hypertension, Diabetes etc.) could this be added to the previous sentence (line 20) that listed what other systematic reviews focused on with an appropriate reference.

Methods:

- Eligibility criteria (line 2-3): Recommend editing “Patients will not be considered eligible if they are under the age of 18 or resident in a nursing home.” to “Patients will be excluded if they are younger than 18 years old.” Will pharmacist interventions in hospital settings be excluded as well? If so, please list as an exclusion criterion. Suggestion to move text currently under the Setting heading, “Clinical pharmacist interventions in a secondary or tertiary care setting will be excluded” to this section.

I confirm that I have read this submission and believe that I have an appropriate level of expertise to confirm that it is of an acceptable scientific standard, however I have significant reservations, as outlined above.

# Response

**Published:** 2020-03-02

**Authors:** Aisling Croke

**Affiliations:** Royal College of Surgeons in Ireland, Ireland

Dear Editors,

Thank you very much for the constructive comments and suggestions received from the editorial team and peer reviewers. We found the suggestions very helpful and hope we have addressed any concerns raised. We have submitted a revised version of the manuscript.

We outline the specific responses to each comment as follows:

**​​​​​​Major Comments:**

**Question 1**

**Main outcomes: Will all of the listed different medication appropriateness criteria (explicit: STOPP/START, Beer’s criteria and implicit: MAI etc.) be used to assess the medication changes of each pharmacist intervention study included in this systematic review? Can the authors please comment on what will determine the use of explicit vs implicit or the use of both explicit and implicit criteria to assess the outcomes of the included studies?**

Author response:

Thank you for highlighting this. It is not the intention in this systematic review to apply different medication appropriateness criteria to details of medications reported in the selected studies. Studies will be included in this review where they use implicit or explicit criteria for medication appropriateness as outcomes as described by study authors. The wording in the main outcomes section on page 6 has been rephrased to clarify that these will be the main outcomes where they have been used within studies.

**Question 2**

**Additional Outcomes:**

**More clarity is needed regarding physical and mental clinical outcomes that will be used as secondary outcome measures. Please list a few specific measures as examples. What are some key clinical outcomes that would be important to consider? (e.g. Falls? Mortality? Blood Pressure? etc.)**

Author response:

The secondary outcome measures of interest in this systematic review have been added to the additional outcomes section on page 6 to give greater clarity.

**Question 3**

**Since the “number of medicines” is mentioned as a major outcome of this systematic review and the introduction discusses deprescribing, is the intention to focus on the reduction of the number of medicines through pharmacist medication/deprescribing medication reviews and reduction of prescribing of high-risk medications that are inappropriately prescribed, or will potential prescription omissions as defined by the START criteria also be applied? It seems the authors intend on the later since medicines optimisation is mentioned under the heading of ‘Review questions’ and in the title of the study. Perhaps defining the concept of optimising medicines as reducing potentially inappropriate prescribing/sub-optimal prescribing via reducing potentially inappropriate medications (PIMs) as defined by Beer’s and STOPP and potential prescription omissions (PPOs) defined by START in the introduction might provide a clearer preface for the main goal of the systematic review.   **     

Author response:             

This systematic review will not apply explicit criteria to outcomes reported by included studies, we will report a narrative synthesis of reported outcomes or a meta-analysis where data allow. We anticipate that studies of medicines optimisation may include different outcomes for medicines optimisation. Some may use number of medicines to assess medicines optimisation, which is a crude measure that does not capture the appropriateness of any medicines stopped or started. Other studies may use reduction in PIMs or PPOs defined using explicit criteria as their measure of medicines optimisation. Within the included studies, reported data on the number of medicines at trial initiation will indicate the degree of polypharmacy in study participants. Where reported at trial end, the number of medicines will capture deprescribing or instances of a missing, clinically indicated medication being commenced. The NICE guidelines on medicines optimisation recommend the adoption of the screening tools as a part of medication review processes, alongside patient-centred shared decision making and appropriate communication with the relevant health care professionals. We have added this information to the introduction section of the paper on page 3.

**Question 4**

**Analysis of subgroups: Inappropriate polypharmacy and inappropriate prescribing is a common problem in older adults. Can the authors please comment on why the cut off chosen here is patients aged 65 years old? For example, is there enough evidence for cost-effectiveness of pharmacist interventions for this age population? Also, if studies that are purely older adult focused (i.e. 65 years and older) are meant to be excluded and are outside the scope of this intended systematic review, please list this explicitly under eligibility/exclusion criteria.**

Author response:

Thank you for clarifying this. Older age is defined by the WHO as people aged 65 years or older (1). Polypharmacy is associated with older age (2), this age group is the population most explicit criteria of prescribing appropriateness apply to, and interventions are typically performed on this age group, as such this was selected as an appropriate cut-off for subgroup analysis. This review will include all adult patients and subgroup analysis will be conducted for the patients younger than 65 years, and those who are aged 65 years and older. The wording in the subgroup analysis section on page 8 has been changed to reflect this clarification. 

**​​​​​​Minor Comments:**

**Question 5**

**Abstract:**

**Methods: Can the authors please clarify what is meant by ‘validated indicators’? Is STOPP/START, Beer’s criteria etc. what is meant? If so, please use screening tools or criteria to assess medication appropriateness instead. Please change in the remainder of the protocol for consistency.**

Author response:

Validated indicators are explicit criteria such as STOPP/START, Beers criteria, or implicit criteria such as the Medicines Appropriateness Index and Prescribing Appropriateness Index. The term validated indicators has been changed to screening tools to ensure consistency.

**Question 6**

**Introduction:   Lines 2-3: “Polypharmacy has commonly been defined as being on five or more medications, however recent studies have highlighted the rising prevalence of more significant polypharmacy defined as being on 10 or more medications”. Whilst I agree with the content of this statement, I would recommend to:**

**1. Refer to more significant polypharmacy (i.e. being prescribed or taking 10 or more medications) as hyperpolypharmacy or excessive polypharmacy as described in the literature.**

**2. Please clarify that polypharmacy overall regardless of which type is on the rise in different age populations.**

Author response:

1. Thank you, we have replaced the term significant polypharmacy with major polypharmacy as described in the literature (3). This review does note excessive and hyper polypharmacy, however major polypharmacy is cited as the most prevalent term.  

2. Text has been inserted into the introduction on page 3 to clarify the prevalence of polypharmacy.

**Question 7**

**Line 3: The referenced article does not mention the term ‘complex polypharmacy’, but rather the complex treatment burden associated with treating comorbidities. Please rephrase to ‘polypharmacy’ to remain consistent with the rest of the introduction.**

Author response:

Text amended to ‘polypharmacy’ as recommended.

**Question 8**

**I would suggest combining the second paragraph beginning with “Polypharmacy is also associated with adverse drug events…” with the earlier paragraph and consolidate main concepts related to the negative consequences of polypharmacy and how polypharmacy is associated with an increased likelihood of experiencing inappropriate polypharmacy.**

Author response:

As per reviewer suggestions we have combined these paragraphs and consolidated the main points on page 3.

**Question 9**

**Line 11: Please define deprescribing.**

Author response:

Deprescribing is the process of withdrawal of an inappropriate medication, supervised by a health care professional (4). This definition has been added to the introduction section on page 3.

**Question 10**

**Line 21: Could the authors please clarify what is meant by this systematic review will not focus on specific conditions? If previous systematic reviews focused on specific conditions (e.g. Hypertension, Diabetes etc.) could this be added to the previous sentence (line 20) that listed what other systematic reviews focused on with an appropriate reference.**

Author response:

We have added text to the introduction section on page 3 to clarify this.

**Question 11**

**Methods:**

**Eligibility criteria (line 2-3): Recommend editing “Patients will not be considered eligible if they are under the age of 18 or resident in a nursing home.” to “Patients will be excluded if they are younger than 18 years old.” Will pharmacist interventions in hospital settings be excluded as well? If so, please list as an exclusion criterion. Suggestion to move text currently under the Setting heading, “Clinical pharmacist interventions in a secondary or tertiary care setting will be excluded” to this section.**

Author response:

We have amended the eligibility criteria on page 4 for clarity as per reviewer comments.

***References:***

*1. Organization WH. Global Health and Ageing on-line: World Health Organization; 2011 [Available from: http://www.who.int/ageing/publications/global_health.pdf.*

*2. Rankin A, Cadogan CA, In Ryan C, Clyne B, Smith SM, Hughes CM. Core Outcome Set for Trials Aimed at Improving the Appropriateness of Polypharmacy in Older People in Primary Care. J Am Geriatr Soc. 2018;66(6):1206-12.*

*3. Masnoon N, Shakib S, Kalisch-Ellett L, Caughey GE. What is polypharmacy? A systematic review of definitions. BMC Geriatr. 2017;17(1):230. **​​​​​***

*4. Reeve E, Gnjidic D, Long J, Hilmer S. A systematic review of the emerging definition of 'deprescribing' with network analysis: implications for future research and clinical practice. Br J Clin Pharmacol. 2015;80(6):1254-68.*
